# Differential impact of government lockdown policies on reducing air pollution levels and related mortality in Europe

**DOI:** 10.1038/s41598-021-04277-6

**Published:** 2022-01-26

**Authors:** Rochelle Schneider, Pierre Masselot, Ana M. Vicedo-Cabrera, Francesco Sera, Marta Blangiardo, Chiara Forlani, John Douros, Oriol Jorba, Mario Adani, Rostislav Kouznetsov, Florian Couvidat, Joaquim Arteta, Blandine Raux, Marc Guevara, Augustin Colette, Jérôme Barré, Vincent-Henri Peuch, Antonio Gasparrini

**Affiliations:** 1 Department of Public Health, Environments and Society, London School of Hygiene and Tropical Medicine, WC1H 9SH London, United Kingdom; 2 European Space Agency, 00044 Frascati, Italy; 3 Centre on Climate Change and Planetary Health, London School of Hygiene and Tropical Medicine, WC1H 9SH London, United Kingdom; 4 European Centre for Medium-Range Weather Forecast, RG2 9AX Reading, United Kingdom; 5 Institute of Social and Preventive Medicine, University of Bern, 3012 Bern, Switzerland; 6 Oeschger Center for Climate Change Research, University of Bern, 3012 Bern, Switzerland; 7 Department of Statistics, Computer Science and Applications “G. Parenti”, University of Florence, 60550 Florence, Italy; 8 MRC Centre for Environment and Health, Department of Epidemiology and Biostatistics, Imperial College London, W2 1NY London, United Kingdom; 9 Royal Netherlands Meteorological Institute (KNMI), 3731 GA De Bilt, The Netherlands; 10 Barcelona Supercomputing Centre, 08034 Barcelona, Spain; 11 Italian National Agency for New Technologies, Energy and Sustainable Economic Development (ENEA), 40129 Bologna, Italy; 12 Finnish Meteorological Institute (FMI), 00560 Helsinki, Finland; 13 A.M. Obukhov Institute for Atmospheric Physics (IAPh), 119017 Moscow, Russia; 14 National Institute for Industrial Environment and Risks (INERIS), 60550 Verneuil-en-Halatte, France; 15 National Center for Meteorological Research (CNRM), University of Toulouse, Météo-France, CNRS, UMR 3589, 31057 Toulouse, France; 16 Centre for Statistical Methodology, London School of Hygiene and Tropical Medicine, WC1E 7HT London, United Kingdom

**Keywords:** Risk factors, Atmospheric chemistry, Environmental monitoring, Scientific data

## Abstract

Previous studies have reported a decrease in air pollution levels following the enforcement of lockdown measures during the first wave of the COVID-19 pandemic. However, these investigations were mostly based on simple pre-post comparisons using past years as a reference and did not assess the role of different policy interventions. This study contributes to knowledge by quantifying the association between specific lockdown measures and the decrease in NO_2_, O_3_, PM_2.5_, and PM_10_ levels across 47 European cities. It also estimated the number of avoided deaths during the period. This paper used new modelled data from the Copernicus Atmosphere Monitoring Service (CAMS) to define business-as-usual and lockdown scenarios of daily air pollution trends. This study applies a spatio-temporal Bayesian non-linear mixed effect model to quantify the changes in pollutant concentrations associated with the stringency indices of individual policy measures. The results indicated non-linear associations with a stronger decrease in NO_2_ compared to PM_2.5_ and PM_10_ concentrations at very strict policy levels. Differences across interventions were also identified, specifically the strong effects of actions linked to school/workplace closure, limitations on gatherings, and stay-at-home requirements. Finally, the observed decrease in pollution potentially resulted in hundreds of avoided deaths across Europe.

## Introduction

COVID-19 disease is caused by severe acute respiratory syndrome coronavirus 2 (SARS-CoV-2). This infectious disease has spread worldwide placing enormous pressure on national health systems since it can cause hospitalization^1^ and lead to death^2^. The first official outbreak was reported in Wuhan (China) in December 2019 and, as of January 4, 2022^3^, the virus is already responsible for 5.46 million deaths worldwide and 1.54 million across Europe. Several local and national policy interventions have been implemented to prevent the transmission of SARS-CoV-2, such as social distancing, stay at home requirements, international travel controls, and non-essential business closures^4,5^. As a consequence of this unique global coordinated response, several urban areas across the world experienced an abrupt drop in air pollution levels^6^. Given the substantive evidence on the short-term effects of air pollution on health, recent studies have suggested that the decrease in exposure of entire populations likely resulted in a reduction in excess mortality and morbidity in different location worldwide^7–10^.

Several studies explored different approaches to assess air pollution changes during the first COVID-19 lockdown. In Europe, Ordóñez and colleagues^11^ used the European Environment Agency's ground monitoring database^12^ to estimate the NO_2_ and O_3_ changes from mid-March to April 2020 compared to 2015–2019. They used a generalised additive model to weather-normalise the daily maximum 1 h mean nitrogen dioxide (NO_2_) and the 8 h mean Ozone (O_3_). They identified an increase of around 10–22% in O_3_ concentrations from northwestern to central Europe based on urban background monitors. Venter et al.^13^ collected satellite and ground station data to estimate air pollution differences between January and May 2020 and a baseline period (2017–2019) in 34 countries. They used a multiple linear regression model to weather-normalise during lockdown period surface concentrations of NO_2_ and particle matter (PM) with an aerodynamic diameter smaller than 2.5 µm (PM_2.5_). They found a reduction in daily mean NO_2_ and PM_2.5_ of 60% and 31%, respectively, with a small increase in ozone (O_3_) of 4%. Following up, Venter et al.^14^ used the estimated changes in pollution to compute the expected reduction in excess mortality and morbidity, reporting a total of 49,900 excess deaths and 89,000 pediatric asthma emergency room visits avoided during the lockdown. Giani et al.^15^ assessed the health impact of daily mean PM_2.5_ concentrations decline in Europe and China by integrating ground station data with a chemical transport model. They simulated the effect of the first COVID-19 lockdown (short-term) on PM_2.5_ concentrations and four emission scenarios of future economic recovery (long-term). In Europe, they found an estimated 2190 short-term avoided deaths (during February–May) and, depending on the economic recovery path, a number of preventable deaths in the long term ranging from 13,600 to 29,500.

Gkatzelis et al.^16^ critically reviewed more than 200 papers and acknowledged the significant effects of meteorological conditions on pollutant concentrations as well as the importance to account for it in the statistical models together with emission trends and atmospheric chemical interactions. They also reported that the majority of publications did not weather-normalise the concentration of pollutants. These steps are relevant because air quality conditions are determined in part by changes in weather and in part by emissions of pollutants from human activities. At different layers of the atmosphere, these two elements will drive a complex package of physical and chemical non-linear interactions^17,18^, determining the spread and concentrations of each pollutant. This makes it complex to conduct simple pre-post comparisons between concentrations in 2020 and previous years. For instance, Barré et al.^19^ demonstrated how much the estimated reduction in NO_2_ levels can vary significantly using simple pre-post or weather-normalised comparisons as well as different data sources. For example, they weather-normalised all estimates and the results demonstrated a large variability on the average reductions from satellite (− 23%) and ground monitors observations (− 43%), and air quality models (− 32%). This study also identified that several previous studies defined the lockdown scenario as a fixed period of low air pollution levels. However, several policy responses were taken by governments at different dates, likely resulting in varying intensity and timing of the reduction in air pollution. More importantly, this simplistic definition prevents the quantitative assessment and comparison of several policy interventions and in particular their differential impact in reducing concentrations of different types of air pollutants.

This study aims first to address all significant effects reported in the literature (i.e. weather correction, emission trends, atmospheric chemistry, and temporally-variant lockdown period of air pollution levels), then to analyse the impact of government responses in reducing the concentration of four pollutants [NO_2_, O_3_, PM_2.5_, and PM < 10 µm (PM_10_)] across 47 European cities (listed in Table 1), and finally to report the related preventable mortality for 46 locations (see “Data and methods”). This study contribute original evidence to the literature by estimating the decline of air pollution levels across Europe in association with the strictness of specific lockdown policies. This assessment will make use of the Copernicus Atmosphere Monitoring Service (CAMS)^20^ operational air quality framework of forecast model ensemble applied under two temporally-variant emission scenarios, representing a business-as-usual (BAU) and lockdown settings, over the exact same period of 2020 (February–July)^21^. The use of innovative data sources and methodological approaches will provide a quantitative assessment of the roles of specific lockdown policy interventions in reducing pollution levels and associated short-term mortality during the study period.

**Table 1:** The sample of 46 cities (except Pristina (Kosovo)) selected from the European CAMS air quality information webpage^42^.

City name	Country	Population	Max SI	Difference in excess deaths by pollutant-specific change (Lockdown-BAU)			
				NO_2_	O_3_	PM_25_	PM_10_
Amsterdam	Netherlands	1,128,715	79.63	− 5.9 (− 7.2; − 4.6)	0.8 (0.6; 1.1)	− 1.8 (− 2.1; − 1.6)	− 1.4 (− 1.5; − 1.2)
Ankara	Turkey	3,002,440	77.78	− 8.3 (− 10.1; − 6.5)	− 1.3 (− 1.7; − 0.9)	− 0.6 (− 0.7; − 0.5)	− 0.7 (− 0.8; − 0.6)
Athens	Greece	3,315,199	84.26	− 40.1 (− 48.8; − 31.2)	− 0.7 (− 1.0; − 0.5)	− 10.0 (− 11.4; − 8.8)	− 7.8 (− 8.7; − 6.9)
Barcelona	Spain	3,832,012	85.19	− 39.2 (− 47.7; − 30.5)	− 1.2 (− 1.6; − 0.8)	− 12.2 (− 13.9; − 10.7)	− 9.3 (− 10.3; − 8.3)
Belgrade	Serbia	1,106,870	100	− 1.6 (− 2.0; − 1.3)	− 2.5 (− 3.2; − 1.7)	− 1.4 (− 1.6; − 1.2)	− 1.0 (− 1.2; − 0.9)
Berlin	Germany	3,271,872	76.85	− 9.6 (− 11.6; − 7.4)	− 1.6 (− 2.1; − 1.1)	− 4.7 (− 5.4; − 4.1)	− 3.5 (− 3.8; − 3.1)
Bern	Switzerland	197,760	73.15	− 0.5 (− 0.6; − 0.4)	− 0.3 (− 0.5; − 0.2)	− 0.5 (− 0.6; − 0.4)	− 0.4 (− 0.4; − 0.3)
Birmingham	United Kingdom	2,426,863	75.93	− 8.9 (− 10.8; − 6.9)	1.0 (0.7; 1.3)	− 4.2 (− 4.8; − 3.7)	− 3.1 (− 3.5; − 2.8)
Bratislava	Slovakia	352,002	87.04	− 1.0 (− 1.2; − 0.8)	− 0.4 (− 0.6; − 0.3)	− 0.5 (− 0.6; − 0.4)	− 0.4 (− 0.4; − 0.3)
Brussels	Belgium	1,381,517	81.48	− 10.4 (− 12.7; − 8.1)	1.4 (0.9; 1.8)	− 3.2 (− 3.7; − 2.8)	− 2.4 (− 2.7; − 2.1)
Bucharest	Romania	1,774,128	87.04	− 9.5 (− 11.5; − 7.4)	− 2.4 (− 3.2; − 1.7)	− 2.8 (− 3.1; − 2.4)	− 2.2 (− 2.4; − 1.9)
Budapest	Hungary	1,758,468	76.85	− 7.3 (− 8.9; − 5.7)	− 2.9 (− 3.9; − 2.0)	− 2.8 (− 3.2; − 2.4)	− 2.3 (− 2.5; − 2.0)
Cologne	Germany	1,508,677	76.85	− 10.1 (− 12.3; − 7.8)	0.6 (0.4; 0.8)	− 4.4 (− 5.0; − 3.8)	− 3.2 (− 3.6; − 2.9)
Copenhagen	Denmark	1,225,959	72.22	− 4.5 (− 5.5; − 3.5)	0.5 (0.4; 0.7)	− 1.2 (− 1.4; − 1.1)	− 1.0 (− 1.1; − 0.9)
Dublin	Ireland	1,004,263	90.74	− 3.3 (− 4.0; − 2.6)	0.3 (0.2; 0.4)	− 1.0 (− 1.2; − 0.9)	− 0.8 (− 0.9; − 0.7)
Hamburg	Germany	1,596,992	76.85	− 7.3 (− 8.9; − 5.7)	0.3 (0.2; 0.4)	− 2.4 (− 2.8; − 2.1)	− 1.9 (− 2.1; − 1.7)
Helsinki	Finland	907,386	60.19	− 1.9 (− 2.3; − 1.5)	0.1 (0.1; 0.1)	− 0.5 (− 0.6; − 0.4)	− 0.7 (− 0.8; − 0.7)
Lisbon	Portugal	1,958,521	87.96	− 18.9 (− 23.0; − 14.7)	0.3 (0.2; 0.4)	− 11.4 (− 13.1; − 10.0)	− 10.6 (− 11.8; − 9.4)
Ljubljana	Slovenia	250,335	89.81	− 0.7 (− 0.8; − 0.5)	− 0.4 (− 0.6; − 0.3)	− 0.4 (− 0.5; − 0.4)	− 0.3 (− 0.3; − 0.3)
London	United Kingdom	9,609,627	75.93	− 37.9 (− 46.1; − 29.5)	4.9 (3.4; 6.5)	− 13.9 (− 15.8; − 12.2)	− 10.5 (− 11.7; − 9.3)
Luxembourg	Luxembourg	119,160	79.63	− 0.4 (− 0.4; − 0.3)	− 0.1 (− 0.1; − 0.1)	− 0.2 (− 0.3; − 0.2)	− 0.2 (− 0.2; − 0.2)
Lyon	France	1,152,368	87.96	− 6.7 (− 8.2; − 5.2)	− 1.0 (− 1.3; − 0.7)	− 2.6 (− 3.0; − 2.3)	− 2.0 (− 2.2; − 1.8)
Madrid	Spain	4,894,295	85.19	− 38.8 (− 47.2; − 30.2)	− 3.4 (− 4.5; − 2.3)	− 7.7 (− 8.7; − 6.7)	− 6.1 (− 6.7; − 5.4)
Marseille	France	909,727	87.96	− 3.2 (− 3.8; − 2.5)	− 1.5 (− 1.9; − 1.0)	− 1.7 (− 1.9; − 1.5)	− 1.3 (− 1.4; − 1.1)
Milan	Italy	3,011,030	93.52	− 36.7 (− 44.7; − 28.6)	− 6.1 (− 8.0; − 4.1)	− 18.1 (− 20.6; − 15.8)	− 12.6 (− 14.0; − 11.2)
Monaco	France	59,433	87.96	− 0.2 (− 0.2; − 0.1)	− 0.2 (− 0.2; − 0.1)	− 0.1 (− 0.2; − 0.1)	− 0.1 (− 0.1; − 0.1)
Munich	Germany	1,573,652	76.85	− 5.5 (− 6.7; − 4.3)	− 1.5 (− 1.9; − 1.0)	− 3.1 (− 3.5; − 2.7)	− 2.3 (− 2.5; − 2.0)
Naples	Italy	3,167,668	93.52	− 29.9 (− 36.4; − 23.3)	− 1.9 (− 2.6; − 1.3)	− 8.2 (− 9.4; − 7.2)	− 5.9 (− 6.5; − 5.2)
Nicosia	Cyprus	228,923	94.44	− 0.3 (− 0.4; − 0.2)	− 0.3 (− 0.4; − 0.2)	− 0.2 (− 0.2; − 0.1)	− 0.1 (− 0.1; − 0.1)
Oslo	Norway	782,172	79.63	− 0.7 (− 0.9; − 0.6)	− 0.1 (− 0.1; − 0.0)	− 0.2 (− 0.2; − 0.2)	− 0.2 (− 0.2; − 0.1)
Paris	France	9,711,652	87.96	− 69.2 (− 84.2; − 53.8)	3.5 (2.4; 4.6)	− 23.2 (− 26.5; − 20.4)	− 17.4 (− 19.3; − 15.4)
Prague	Czech Republic	1,126,681	82.41	− 2.6 (− 3.2; − 2.1)	− 1.0 (− 1.3; − 0.7)	− 1.6 (− 1.8; − 1.4)	− 1.1 (− 1.2; − 1.0)
Pristina	Kosovo	196,913	92.59	NA (NA; NA)	NA (NA; NA)	NA (NA; NA)	NA (NA; NA)
Reykjavik	Iceland	184,357	53.7	− 0.1 (− 0.1; − 0.1)	0.0 (0.0; 0.0)	− 0.0 (− 0.0; − 0.0)	− 0.0 (− 0.0; − 0.0)
Riga	Latvia	556,672	65.74	− 0.6 (− 0.8; − 0.5)	− 0.4 (− 0.5; − 0.3)	− 0.4 (− 0.4; − 0.3)	− 0.3 (− 0.3; − 0.3)
Rome	Italy	2,342,860	93.52	− 18.4 (− 22.4; − 14.3)	− 5.8 (− 7.7; − 4.0)	− 6.8 (− 7.7; − 5.9)	− 4.8 (− 5.3; − 4.3)
Sarajevo	Bosnia and Herzegovina	371,884	92.59	− 0.4 (− 0.5; − 0.3)	− 0.7 (− 1.0; − 0.5)	− 0.3 (− 0.4; − 0.3)	− 0.2 (− 0.3; − 0.2)
Sofia	Bulgaria	926,881	73.15	− 3.5 (− 4.3; − 2.7)	− 1.4 (− 1.9; − 1.0)	− 1.1 (− 1.3; − 1.0)	− 0.8 (− 0.9; − 0.8)
Stockholm	Sweden	1,305,076	46.3	− 1.8 (− 2.2; − 1.4)	− 0.2 (− 0.3; − 0.1)	− 0.7 (− 0.8; − 0.6)	− 1.1 (− 1.2; − 0.9)
Tallinn	Estonia	344,511	77.78	− 0.5 (− 0.7; − 0.4)	− 0.1 (− 0.1; − 0.1)	− 0.1 (− 0.2; − 0.1)	− 0.1 (− 0.1; − 0.1)
Tirana	Albania	719,252	89.81	− 1.5 (− 1.8; − 1.1)	− 0.9 (− 1.1; − 0.6)	− 0.7 (− 0.7; − 0.6)	− 0.5 (− 0.6; − 0.4)
Turin	Italy	1,205,385	93.52	− 13.3 (− 16.2; − 10.3)	− 3.6 (− 4.8; − 2.5)	− 6.8 (− 7.8; − 6.0)	− 4.9 (− 5.4; − 4.3)
Valencia	Spain	1,393,120	85.19	− 8.0 (− 9.7; − 6.2)	− 1.9 (− 2.6; − 1.3)	− 3.3 (− 3.7; − 2.9)	− 2.5 (− 2.8; − 2.2)
Vienna	Austria	1,856,676	85.19	− 6.1 (− 7.4; − 4.7)	− 1.8 (− 2.4; − 1.2)	− 3.1 (− 3.5; − 2.7)	− 2.3 (− 2.5; − 2.0)
Vilnius	Lithuania	355,430	87.04	− 0.8 (− 1.0; − 0.6)	− 0.2 (− 0.3; − 0.1)	− 0.3 (− 0.4; − 0.3)	− 0.2 (− 0.3; − 0.2)
Warsaw	Poland	1,789,294	83.33	− 7.5 (− 9.2; − 5.9)	− 1.0 (− 1.3; − 0.7)	− 2.9 (− 3.3; − 2.5)	− 2.0 (− 2.2; − 1.8)
Zagreb	Croatia	660,653	96.3	− 1.9 (− 2.3; − 1.4)	− 1.5 (− 1.9; − 1.0)	− 1.2 (− 1.4; − 1.1)	− 1.0 (− 1.1; − 0.9)
TOTAL		82,555,333	100	-485.5 (− 590.9 ; -377.6)	− 36.5 (− 57.1; − 16.0)	− 174.6 (− 199.0; − 153.1)	− 133.5 (− 148.2; − 118.3)

Reported are the population, the maximum daily *Stringency Index *(SI) reached in each city, and the estimated difference in number (with credible limits) of excess deaths associated with the change (Lockdown–BAU difference) in the four pollutants concentration. Negative values indicate that avoided deaths were expected from the Lockdown-BAU difference.

## Data and methods

### Data

#### Design setting

This study originally selected 50 cities among the largest in Europe to represent most of the countries and populations (Table 1)^4^. Among these 50, three were excluded (Podgorica—Montenegro, Skopje—Moldova, and Valletta—Malta) because of the absence of government response data. This study reported the results of the government responses to lower air pollution for 47 cities; however, the excess deaths were estimated for 46 cities since the mortality rate for Pristina (Kosovo) was not available on the Eurostat database^22^. The study period ranges from 1st of February to 31st of July 2020 and roughly represents the first wave of COVID-19 pandemic in Europe, with a short initial period characterised by the absence of government responses, followed by a strict implementation and then partial relaxation of lockdown policies. For this period and cities, concentrations of four air pollutants (NO_2_, O_3_, PM_2.5_, and PM_10_) were extracted from numerical forecast models (see below) under two emission scenarios: BAU and Lockdown^21^.

#### Numerical forecast model dataset

CAMS is one of the services that form Copernicus, the European Union's Earth observation programme providing quality-controlled information related to air pollution via chemical transport models that are driven by a single numerical weather prediction model. An ensemble of six state-of-the-art chemistry-transport numerical forecast models (Table A3) included in the CAMS continuous air quality monitoring service has been used to simulate air pollution concentrations under the two emission scenarios. The BAU scenario considers no government restrictions, with air pollution emissions running at their default inventory demand. The BAU emissions are obtained from the CAMS-REG-APv4.2 gridded inventory (0.1 × 0.05°)^23^ constructed by Kuenen et al.^24^, which is largely based on the official reported emission data from individual countries in Europe to the Centre on Emission Inventories and Projections (CEIP) at European Monitoring and Evaluation Programme (EMEP) for each source category. The Lockdown scenario is based on daily-, sector-, pollutant- and country-dependent emission reduction factors determined by the Barcelona Supercomputing Centre^21^. The rationale for the reduction factors was supported by many relevant activity-based and open-access observations, for example, Google mobility reports^25^. The CAMS-REG-APv4.2 inventory was combined with the emission reduction factors in order to model dynamic emission reductions for each sector and country in the Lockdown scenario. The base year of the CAMS-REG-APv4.2 emissions used in the two scenarios was 2017, which was the most recent year available at the time of the study. Meteorological forcing that was used to generate the simulation remained identical, enabling a consistent comparison between the two scenarios. This CAMS product provides hourly concentrations of the ensemble median of the six models at the surface level of each pollutant across Europe in a regular latitude–longitude grid of 0.1° (approx. 10 × 10 km^2^). This study extracted for each of the 47 cities daily averages of Lockdown–BAU difference for NO_2_, PM_2.5_, and PM_10_ and daily maximum 8 h mean for O_3_.

#### Oxford coronavirus government response tracker

The Oxford Coronavirus Government Response Tracker (OxCGRT)^4^ dataset systematically collects information on governments responses to the COVID-19 pandemic. This global dataset is updated daily with information translated into 19 individual policy measures that are classified in four groups: containment and closure policies (C), economic policies (E), health system policies (H) and miscellaneous policies (M). These individual measures are coded as an integer between 0 (no government measure) and a maximum level that depends on the measure (usually between 2 and 4). The OxCGRT dataset also proposes several thematic indices combining subsets of the 19 measures.

This study focused on a quantitative measure of the strictness of the lockdown interventions, the *Stringency Index* (SI). This measure is constructed as the mean of nine policy measures: all eight C policies and the H1 policy that records public information campaigns (Table A1^4^). The SI is computed as the mean of the standardized policy measures (between 0 and 1), so that each policy measure contributes equally to the SI, independently from its number of levels. The SI is then rescaled to have values between 0 (no response) and 100 (maximum response in every possible policy measure).

#### Health impact assessment

For the health impact assessment, estimates of relative risk (RR) of mortality associated with short-term exposure to each pollutant were collected from published multi-country studies for NO_2_^26^, O_3_^8^, PM_10_^9^ and PM_2.5_^9^. In addition, city-specific variables potentially related to differential pollution levels, specifically population size, NDVI (greenness), and built-up area, were collected for the year 2015 from the Global Human Settlement Urban Centres Database (GHS-UCDB)^27^. Finally, city-specific crude all-cause mortality rates for 2015 were extracted from Eurostat^22^.

### Statistical analysis

The statistical analysis follows three main steps: (1) estimation of the association between SI and changes in pollution between Lockdown and BAU scenarios, (2) estimation of the specific impact of each sub-policy measure on the pollution change, and (3) health impact assessment with the quantification of avoided deaths due to short-term exposure to air pollution. Each of these steps was performed independently for each of the four pollutants. The analysis was performed in R-4.0.3^28^ with the addition of the integrated nested Laplace approximation (INLA) package^29^.

#### Association between Stringency Index and pollution difference

The first step of the study modelled the association between changes in air pollution and SI through a spatially structured Bayesian non-linear mixed effect model, expressed by Eq. (1):

1 $${y}_{it}=f\left({x}_{it};{\varvec{\beta}}+{{\varvec{b}}}_{i}\right)+{{\varvec{\gamma}}}_{1i}{DOW}_{t}+{\gamma }_{2}NDV{I}_{i}+{\gamma }_{3}{BuiltUp}_{i}+{\epsilon }_{it}$$

where ${y}_{it}$ represents the pollutant-specific change (Lockdown-BAU) for city *i* and day *t*; ${x}_{it}$ is the daily SI for the city *i*; *DOW* represents a factor for day-of-week with city-specific coefficients ${{\varvec{\gamma}}}_{1i}$, while *NDVI* and *BuiltUp* area are city-specific indicators with related coefficients ${\gamma }_{2}$ and ${\gamma }_{3}$. The ${{\varvec{\gamma}}}_{1i}$ is estimated as city-specific since the DOW effect magnitude varies from city to city. ${\epsilon }_{it}$ is an unstructured Gaussian residual. Note that the intercept is removed from the model since no pollutant change (${y}_{it}=0$) is expected in the initial phase of no government response.

The nonlinear term $f\left({x}_{it};{\varvec{\beta}}+{{\varvec{b}}}_{i}\right)$ is expanded through natural splines with four degrees of freedom and three knots placed at the 25, 50, and 75% quantiles. ${\varvec{\beta}}$ is thus a four-dimensional vector representing fixed effect and ${{\varvec{b}}}_{{\varvec{i}}}$ are four-dimensional vectors representing city-specific deviations from the fixed effects ${\varvec{\beta}}$. For each of the four coefficients in ${{\varvec{b}}}_{i}$, a spatial structure is added through a stochastic partial differential equation (SPDE) approach^30^. Specifically, a Matérn covariance function was applied with penalized complexity prior as defined by Franco-Villoria et al.^31^.

The model in Eq. (1) is fitted through the INLA procedure for Bayesian estimation^32^. Uninformative flat priors were used for all unstructured coefficients. In the main manuscript body, this study reports posterior means and 95% credible intervals of predicted pollutant changes for a range of SI between 0 and 80%, as higher levels were not observed in most cities.

#### Impact of specific policy measures

The second step of the study assessed the impact of specific policy measures. Each policy indicator used to compute the SI was included as a new variable in the model of Eq. (1), generating the new following Eq. (2):

2 $${y}_{it}=\left(\mathrm{\alpha }+{a}_{\mathrm{i}}\right){z}_{\mathrm{it}}+f\left({x}_{it}^{^{\prime}};{\varvec{\beta}}+{{\varvec{b}}}_{i}\right)+{{\varvec{\gamma}}}_{1i}{DOW}_{t}+{\gamma }_{2}NDV{I}_{i}+{\gamma }_{3}{BuiltUp}_{i}+{\epsilon }_{it}$$

where ${z}_{it}$ is the policy measure with associated fixed and city-specific effects $\alpha$ and ${a}_{i}$, and ${x}_{it}^{^{\prime}}$ is the SI measure with ${z}_{it}$ removed. We standardize ${z}_{it}$ so that it takes zero when the policy is not implemented and one at its maximum level of stringency. The coefficient $\left(\mathrm{\alpha }+{a}_{\mathrm{i}}\right)$ then represents the effect of the policy’s maximum level. All other components in Equation Eq. (2) are the same as their counterparts in Equation Eq. (1). As for the analysis of the full SI, city-specific policy coefficients ${a}_{i}$ are also spatially structured with a Matérn covariance function and corresponding penalized complexity priors. The model in Eq. (2) is also fitted by INLA. “Results” section reports posterior means of $\alpha$ for each pollutant and policy measure.

### Estimating the excess deaths attributable to changes in air pollution levels

Given the estimates of the association between each pollutant and mortality $\xi$, daily mortality burdens are computed as:

3 $${d}_{it}={m}_{i}\times {p}_{i}\times (1-{e}^{-\widehat{\xi }{y}_{it}})$$

where ${m}_{i}$ is the crude death rate, ${p}_{i}$ the population of city $i$ and ${y}_{it}$ the observed pollutant difference. $\widehat{\xi }$ is the pollutant-specific effect on mortality gathered from the literature^8,9,26^. The estimated values of ${d}_{it}$ are then summed by city to obtain total city-specific mortality burdens for the period of February to July. To compare results, Table A4 reports both the total deaths by city and the excess deaths under the BAU scenario by pollutant and city, both during February and July 2020.

The uncertainty assessment of the mortality burdens was obtained by parametric Monte-Carlo simulations. A total of 1000 values were sampled from a Gaussian distribution with mean $\widehat{\xi }$ and standard deviation derived from the confidence intervals reported in the literature, and for each simulated coefficient, compute the mortality burden. Empirical confidence intervals are then obtained by computing the percentile 2.5th and 97.5th of a sample of 1000 iterations of computed mortality burdens.

## Results

### Analysis of changes in air pollution levels across Europe

**Figure 1:**
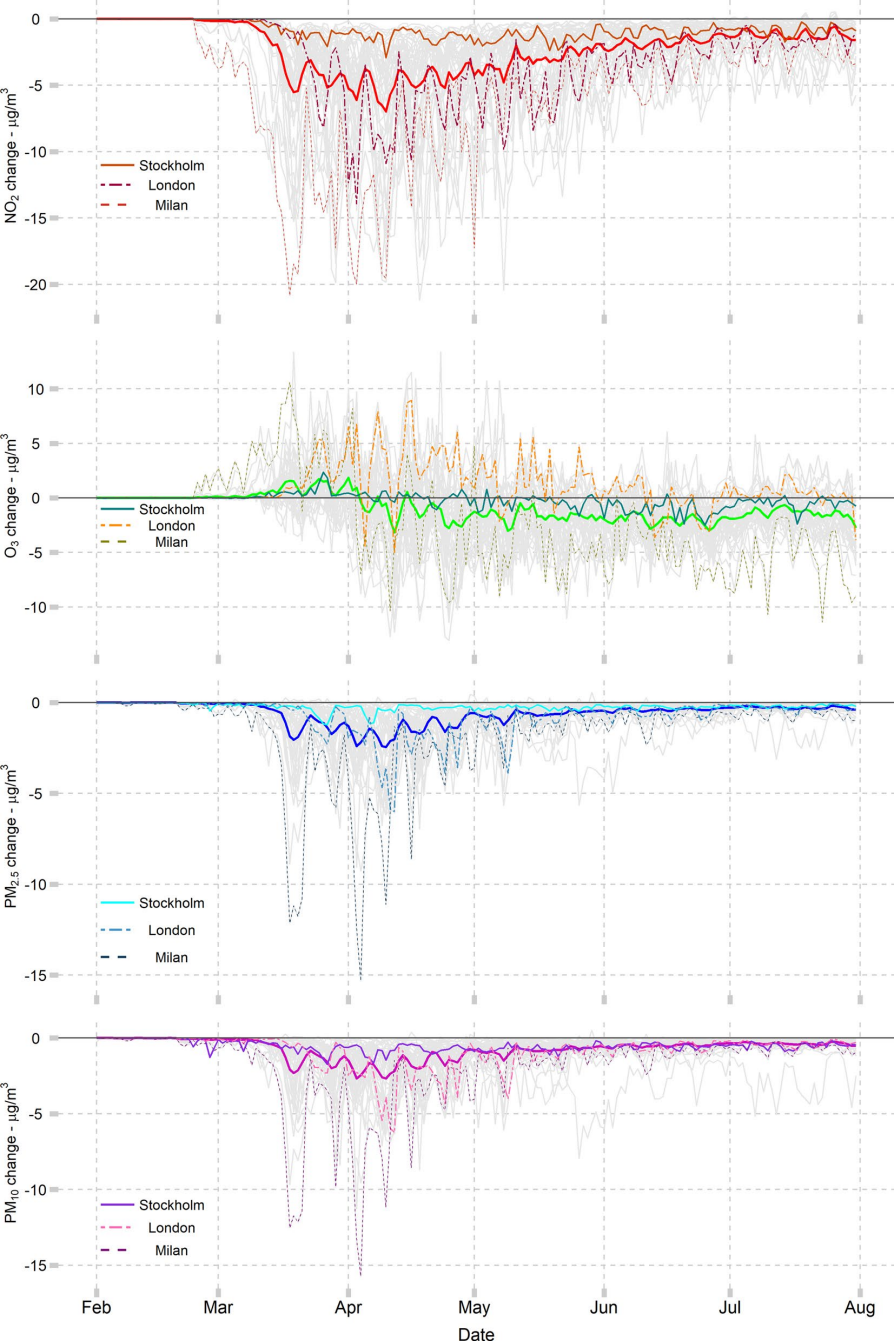
Pollutant change represented as % (Lockdown–BAU differences). NO_2_ and PM are expressed by daily mean and O_3_ by daily maximum 8 h-mean. This study includes 47 cities (solid thin light grey lines) and their average (solid thick coloured line) from 1st February to 31st July 2020. Three cities [Stockholm (Sweden), London (United Kingdom), and Milan (Italy)] were displayed with solid thin, dashed, and twodash coloured patterns, repectively. Figure created using R software, version 4.0.3^28^.

Several of the atmospheric models that are part of CAMS regional air quality forecast system were used to simulate the concentrations of the four pollutants under two scenarios, defined as ‘Lockdown’ and ‘BAU’ during the same period of 2020 (February–July). Their differences were used in this study to estimate city-specific changes in the daily concentration of each pollutant type. Figure 1 displays the time series of the change in levels of the daily mean of NO_2_ and PM and daily maximum 8 h mean for O_3_ in each of the 47 cities (listed in Table 1) and their average. Plots of NO_2_ and PM indicate that their concentrations started to plummet during the first half of March, when the government responses were first implemented across the majority of the European cities. Differences across cities can be related to the different timing of lockdown policy implementations, as well as variation in strictness and potential effects of the policies. For instance, Milan (Italy) shows an earlier decline compared to the other cities, until the NO_2_ concentrations dropped to their minimum around mid-March. In contrast, London (United Kingdom) experienced a noticeable decrease only in the second half of March. Stockholm (Sweden) had its air pollution levels less affected during the study period due mostly to the less stringent interventions from the government. After the strong decline in March/April, all cities experienced an attenuation in their NO_2_ and PM changes, but still keeping their levels lower than in the BAU scenario. Concentrations of daily maximum O_3_ show a different pattern compared to the other three pollutants. The relative changes are very limited throughout the period with a slight increase up to the end of April, followed by very limited reductions for the remainder of the period. The seasonal variability of O_3_ is very different compared to other pollutants because of its specific photochemical sensitivity which leads to enhanced formation in the summer season, while reductions in nitrogen oxides emissions (NO_x_, combination of nitrogen oxides NO and NO_2_) can lead to an increase of O_3_ as a result of reduced titration and/or depending on the mix of O_3_ production precursors (NO_x_ and VOCs). Titration is of particular concern close to emission sources, at night, or in winter, in part due to conditions of increased atmospheric stability, but it can occur over a wide range of conditions. The specific temporal pattern of O_3_ shown in Fig. 1 is therefore likely attributed to the transitioning between winter and summertime chemistry in the April/May period.

### Quantifying the effect of lockdown strictness

Changes in pollution levels were then linked with measures of government policy responses to the pandemic, collected from the OxCGRT^4^ (Table A1). In addition to offer a systematic definition of lockdown measures, the database provides quantitative indices of strictness across countries and periods. Specifically, an overall measure is offered by the SI (ranging from 0 to 100%), a summary of nine indicators related to containment, closure, and health policies. Table 1 reports the maximum daily SI score across the sample of cities, while Figure A1 in the supplementary material illustrates the average and city-specific daily series. As expected, there is a common temporal pattern in the strictness of the implemented policies, although with noticeable differences across cities. Until early March, many cities experienced an increase in SI level by modest steps when an abruptly jump to an SI level around 75% was seen for most of the locations, and by mid-May, the SI levels were then generally going down by large steps (Figure A1). Belgrade (Serbia) was the only city having the maximum government response index (100%) while Stockholm (Sweden) reached only 46.3% (Table 1).

A quantitative estimate of the association between the strictness of lockdown policies and decrease in air pollution was obtained by relating daily Lockdown–BAU differences to SI measures in each of the 47 cities. The association was estimated based on a Bayesian hierarchical spatio-temporal model implemented using the INLA method. The model used daily time series of change in each pollutant as the outcome and the SI as a predictor, while controlling for the day of the week, normalised difference vegetation index (NDVI), and built-up area. This advanced methodology allowed us to flexibly model city-specific non-linear exposure–response relationships and to account for spatial correlation across locations in Europe.

**Figure 2:**
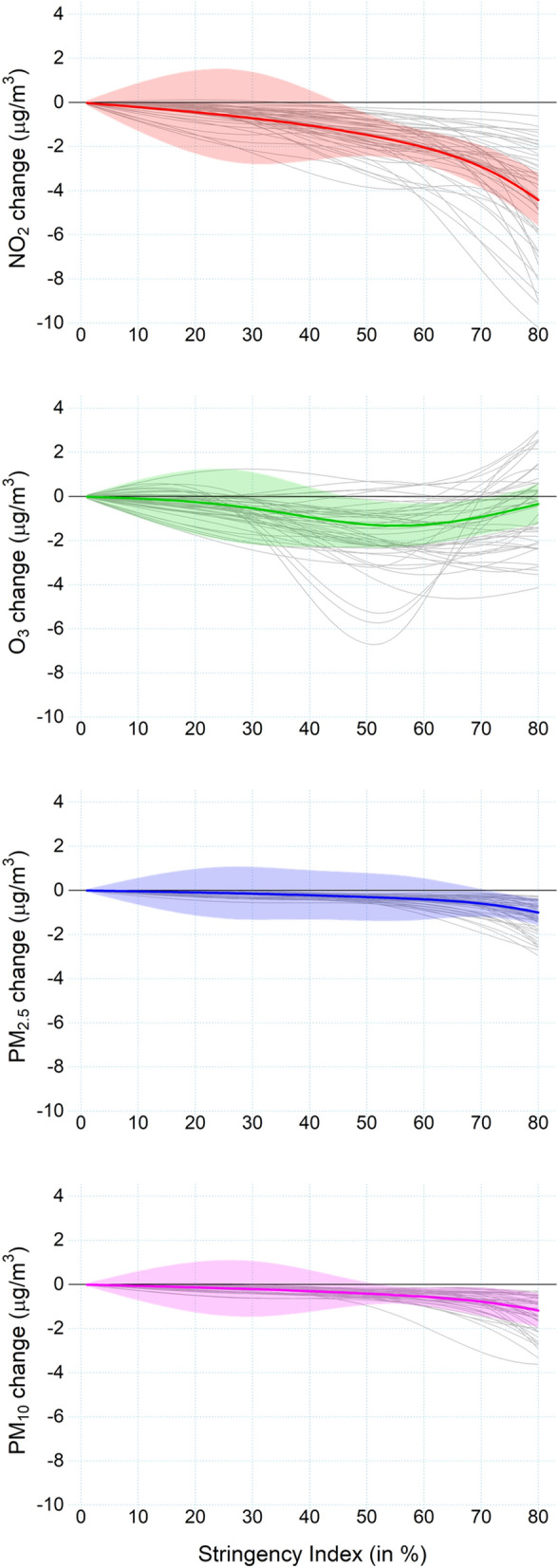
Estimated association between the SI score and change (Lockdown–BAU differences) for each pollutant. NO_2_ and PM are expressed by daily mean and O_3_ by daily maximum 8 h-mean. All 47 cities are represented by thin light grey lines, with the average as the thick coloured line. The coloured shaded area represent the credible intervals of the average effect. Figure created using R software, version 4.0.3^28^.

Figure 2 displays the results as average and city-specific estimated change in concentrations across the SI range. On average, results indicate an inverse association for all cities, showing a decrease when increasing the strictness of government policies. This decrease is mild for low values of SI but sharpens at higher values. The decrease was substantial for NO_2_, while the PM shows a weaker effect. Results for O_3_ suggest a lower effect at very high SI ranges. The different relationship with O_3_ can be influenced by the more complex temporal pattern of this pollutant and the role of seasonal factor that determine its concentration. The wider 95% credible interval seen between 20 and 40% SI in Fig. 2 is likely due to the sparse data within this range, as most of the cities have recorded levels jumping from around 0 to very high values.

**Figure 3:**
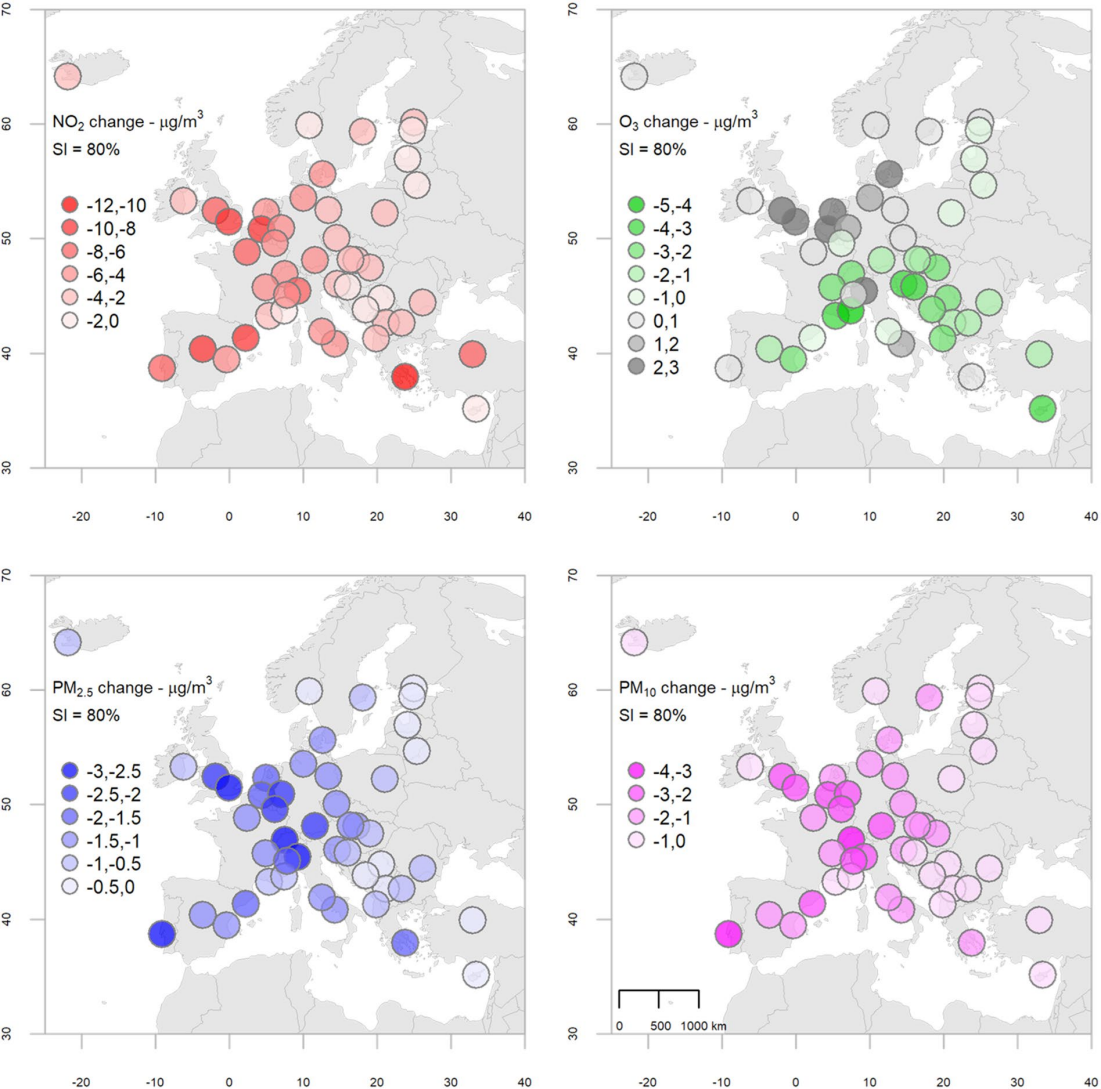
Change in each pollutant’s concentration estimated at 80% SI score across the 47 cities in Europe. NO_2_ and PM are expressed by daily mean and O_3_ by daily maximum 8 h-mean. Figure created using R software, version 4.0.3^28^.

Figure 3 displays maps of changes in each pollutant predicted with an 80% SI score (a value reached in most of the cities) across the European region. The actual estimates are reported in full in Table A2, together with 95% credible limits. The maps suggest a clear geographical pattern, although with some differences between pollutants. The strongest effect on NO_2_ for an 80% SI score was seen in Athens (Greece), presenting a Lockdown–BAU difference around − 10.2 µg/m^3^ (− 10.7; − 9.7) (Table A2). London shows strong effects, with a increase for O_3_ of 2.9 µg/m^3^ (1.9; 4.0) and a decrease of − 9.0 µg/m^3^ (− 10.1; − 7.9), − 2.6 µg/m^3^ (− 3.0; − 2.2), and − 2.9 µg/m^3^ (− 3.4; − 2.5) for NO_2_, PM_2.5_, and PM_10_, respectively. A clear latitudinal gradient is found for O_3_, because of its specific photochemical formation, where titration effects are more pronounced in northern NO_x_ saturated areas such as the Benelux region, whereas reductions are found around the Mediterranean.

**Figure 4:**
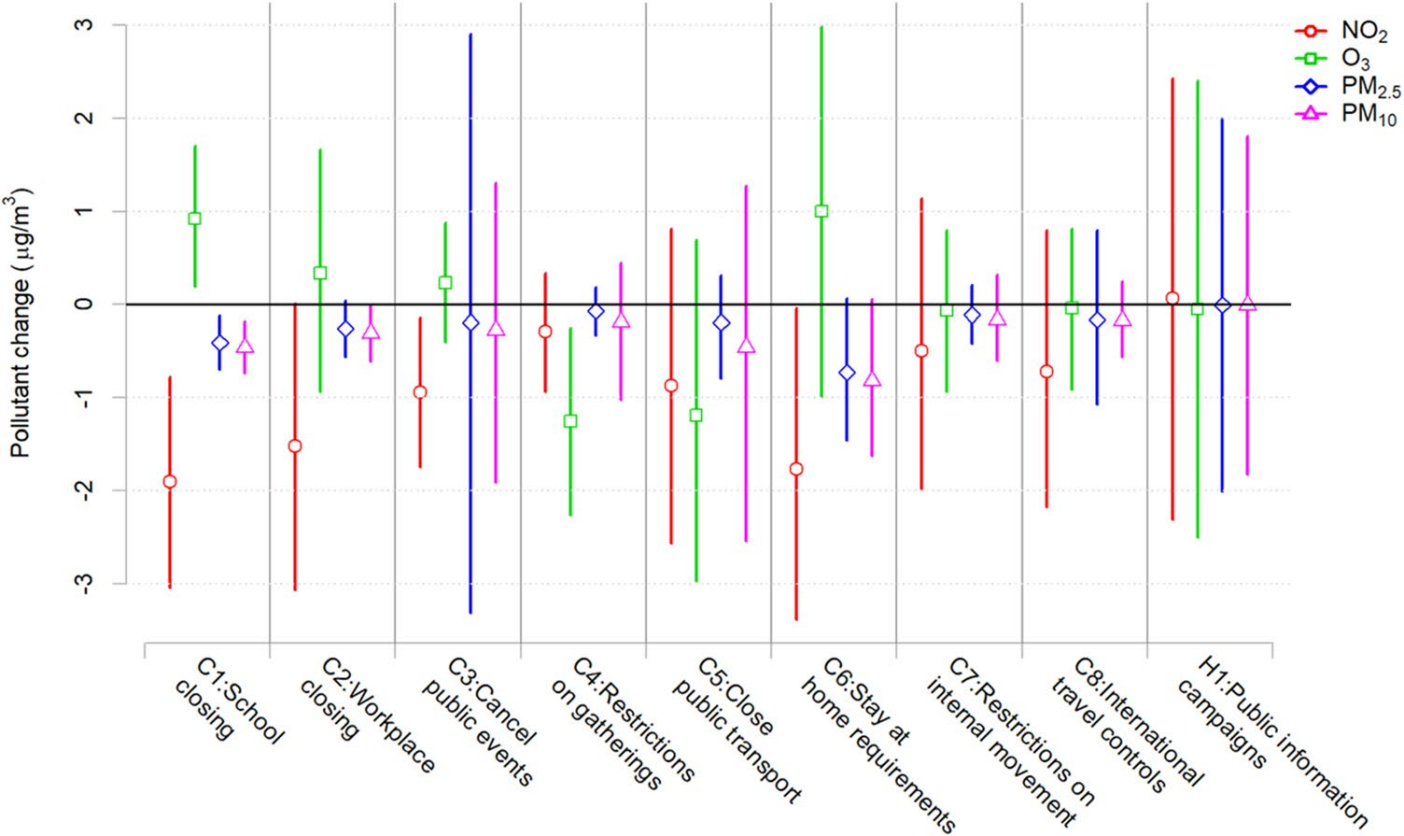
The effect of individual policies that compose the SI score on changes in the four pollutants (Lockdown–BAU differences), with 95% credible intervals. Figure created using R software, version 4.0.3^28^.

The third part of this study focused on investigating the contribution of individual policy indicators used to compute the SI on the reported change in air pollutants. The strictness levels of each indicator are described in Table A1. In this part, separate models were fitted to assess policy indicators individually, using each of them as a predictor along with the SI computed without it, thus disentangling their respective contribution. Results reported in Fig. 4 show that decreases in NO_2_ were mostly linked with policies that limited the daily commute travels, such as C1 (school closing), C2 (workplace closing), C3 (cancel public events), and C6 (stay at home requirements). Results for PM_2.5_ and PM_10_ show a consistent pattern, although with lower effects. C1 and C3 policies had a strong effect in increasing O_3_ concentrations during lockdown scenario (with similar but more uncertain evidence for C2 and C6), while C4 (restrictions on gatherings) contributed to lower O_3_ levels. Interesting to note is that some policies, such as C7 (restriction on internal movement), C8 (international travel controls), and H1 (public information campaigns) seemed to have little impact on pollution concentrations.

### Avoided mortality due to short-term exposure to air pollution

The fourth and last part was to estimate the number of premature deaths avoided due to the decline in air pollution levels associated with the government responses. The total deaths were estimated for each city using exposure–response relationships reported in recent literature for each pollutant and the observed changes in daily concentration, independently of the SI levels. Results are reported in Table 1, indicating a total number of avoided deaths of 486, 37, 175, and 134 for NO_2_, O_3_, PM_2.5_, and PM_10_ across the 46 cities [except Pristina (Kosovo)]. Paris (France), London, and Barcelona (Spain), and Milan are within the top six cities with the highest number of avoided deaths for NO_2_ and PM (Table 1). However, the highest excess deaths for O_3_ were found in London and Paris.

## Discussion

The response of governments to curb the COVID-19 pandemic spread offers an unprecedented case study to assess a range of interventions to reduce anthropogenic emissions from several sectors (i.e. road transport, energy industry, manufacturing industry, commercial and public services, shipping, and aviation). In this context, this study contributes to the literature in many aspects. First, it provides an accurate representation of air pollution decline during the first pandemic phase that was simulated by comparing lockdown versus BAU scenarios using an ensemble of six state-of-the-art numerical forecast models from the CAMS. The changes in NO_2_, O_3_, PM_2.5_, and PM_10_ were then separately regressed against the SI levels (i.e. standardized lockdown measures), thus providing quantitative estimates of the association with the strictness of the policies. The estimation was performed using a spatio-temporal Bayesian non-linear mixed effect model. This advanced methodological approach can estimate flexible relationships across cities while accounting for spatial dependencies. The main original aspect of this study is the assessment of multiple indices related to individual lockdown policies, in order to evaluate their comparative role in determining changes in air pollution. Finally, a quantitative health impact assessment was performed for the period of February–July of 2020, estimating the avoided/excess deaths due to the air pollution changes in 46 of the 47 European cities.

It has been reported by several space^33^ and environment agencies^34^ that air quality satellites and ground-based monitors captured an abrupt drop in air pollution levels in several cities during the first COVID-19 lockdown. The literature includes many studies which have compared pollutant concentrations before and after the start of the lockdown or comparing the year 2020 to another. These procedures can neither account for the influence of weather variability nor account for complex atmospheric processes and chemical interactions of multiple pollutants and precursors. This study innovates from published approaches by using CAMS^20^, an ensemble atmospheric model, which simulates pollutant concentrations in two scenarios during the exact same period and identical weather conditions. The quality of the CAMS regional production is closely monitored with regular quality control^35^ and the models are also used in support of European air quality policies^36^. The results demonstrated that NO_2_ was the pollutant with the largest decline (Fig. 1) displaying a reduction above 50% for Spain (Madrid), Portugal (Lisbon), France (Lyon and Paris), and Italy (Milan, Turin, and Rome). Although satellite, ground-observations, and modelled-based estimates demonstrate large discrepancies, Barré and colleagues^19^ using weather-normalised estimates found a consistent decrease of NO_2_ surface concentrations across Europe. They also highlighted Spanish, Italian and French cities as the locations with the largest effect (around 50–60%). The main reason for NO_2_ noticeable drop is because its main emission contributor (road transport) was the most affected sector by government restrictions. This study presented a smaller mean reduction in PM_2.5_ of 0.60 µg/m^3^, compared to Giani et al.^15^ (1.82 µg/m^3^) using a shorter period (from 21st February to 17th May). However, they described PM_2.5_ peaks of − 6.6 µg/m^3^ while this study found higher PM_2.5_ change extremes (i.e., − 15.30 µg/m^3^). The PM_2.5_ reductions are likely associated with policy interventions also in the energy, manufacturing industry, and commercial sectors; however, a small increase should be considered from residential activities due to stay-at-home requirements. In contrast, ground-level O_3_ concentrations increased slightly in urban locations across Europe at the beginning of the lockdown (April), primarily as a result of the absence or a lower titration of O_3_ by NO_x_ emissions from industrial and motor vehicle activities^37^. Ordóñez and colleagues^11^ identified higher O_3_ concentration during March–April 2020 compared to March–April 2015–2019 mostly over the northwestern area (i.e. Benelux region and the United Kingdom). They emphasized the role of meteorological variability in this comparison of an individual year (2020) with a 4-year average of the previous period. During March–April 2020 over the same area, this study also detected a high increase in O_3_ production compared to the BAU scenario. However, thanks to the use of a fixed meteorological year in our approach (i.e., both Lockdown and BAU scenarios performed during March–April 2020), this study can conclude that this O_3_ increase was mainly due to the reduced NO_x_ concentrations and exclude meteorological factors. On the other hand, Ordóñez and colleagues results on the decrease in O_3_ changes over Portugal and Spain contrasted with the increasing O_3_ changes found in this study.

To our knowledge, this is the first study that quantifies the decline of air pollution levels across Europe in association with the strictness of specific lockdown policies. This analysis is based on flexible statistical techniques to capture effects across the range of standardised policy indices. The findings also revealed clear evidence of non-linear relationships, with stronger changes at higher SI values, indicating that stricter lockdown policies were more effective in decreasing air pollution. Extending the assessment to individual policies, this study revealed that the reduction in pollutant’s concentration cannot be attributed to all policies included in the SI. Government actions linked to school/workplace closure, limitations on gatherings and stay-at-home requirements had the greatest impact on reducing NO_2_ concentrations. This is likely explained by their effectiveness in limiting local mobility, therefore, reducing the large contribution of road transport emissions to the total NO_x_ primary emissions^38^. Despite the large drop in road transport emissions (one of the primary and secondary PM precursor emission sources), PM levels were reduced more modestly since they are also produced by natural sources. The secondary component of PM, which can respond non-linearly to emissions, may also be behind this moderate response, while some cities experienced a slight PM increase due the stay-at-home requirements which can stimulate the increase of residential wood combustion. However, even if wood combustion represents over half of PM primary emissions^39^, its contribution to the total PM emissions was very limited during the first lockdown wave^40^ due to the season period and its usage purpose (indoor heating) across Europe. The results also demonstrated that policies banning national movements and international travels seem less successful in lowering air pollution. This restriction relates mainly to emissions from the aviation sector, which usually have a low contribution to the overall urban air quality levels. These findings can contribute to the definition of future European strategies and priorities on the design and implementation of policies for reducing air pollution levels in urban areas. As a public health measure, this reduction experienced across Europe during February-July of 2020 could have prevented hundreds of deaths associated with short-term exposure to air pollution. Based on independent estimates for NO_2_, O_3_, PM_2.5_, and PM_10_, it could have avoided 486, 37, 175, and 134 deaths (Table 1) compared to 2,573, 5,190, 2,441, and 2,186 excess deaths estimated under a BAU scenario (Table A4) respectively, over the same period.

This study also faced some limitations that must be acknowledged. This study did not investigate the following lockdown waves across Europe after July 2020 because the concentration of pollutants for the lockdown scenario were provided only between February and July 2020. The strictness of government policies (expressed by the SI) corresponds to the country’s response rather than city-specific interventions. The wide confidence intervals in Fig. 4 suggest that there is still important uncertainty about the results, stemming from the important collinearity between the individual policies that were roughly implemented at the same time. Therefore, such evidence should not be straightforwardly used to define specific public policies since it must be based on a broader assessment of the literature and not on the results of a single study. In the health impact assessment, this study applied the pollutant’s exposure–response relationship representing the average value reported in the referenced papers; therefore, not accounting for potential heterogeneity in the associated health risks across cities. The excess deaths also should be interpreted with caution, since all locations experienced a decrease in concentrations for many pollutants at the same period, with the NO_2_﻿-, O_3_-, PM_10_-, and PM_2.5_-related avoided deaths partially overlapping. However, previous studies reported^41^ a low risk of double counting, with similar associations between pollutant and deaths from single-pollutant (e.g. NO_2_^10^) and two-pollutant concentration–response models (e.g. NO_2_ and PM_2.5_^9^). Another important point to acknowledge is that the preventable deaths reported in this study were estimated using outdoor air pollution levels, while lockdown conditions forced most of the people to spent most of their time indoors. Finally, this study did not account for cause-specific deaths or other health outcomes, as well as long-term mortality risks. This will be likely addressed in future research.

To conclude, this study assessed the association between standardised measures of global and individual policies responses to the COVID-19 pandemic with changes in air pollution and short-term premature mortality in Europe. These findings provide evidence on the effectiveness of government restrictions and target policies for reducing air pollution concentrations in urban areas and demonstrate the public health benefits of reducing human exposure to high air pollution levels across Europe.

## Supplementary Information

Supplementary Information.
