# Supplementary material for: Differential impact of government lockdown policies on reducing air pollution levels and related mortality in Europe
Source: Sci Rep. 2022 Jan 26;12:726. doi: 10.1038/s41598-021-04277-6 (PMC8791935; doi:10.1038/s41598-021-04277-6)
Supplement: Supplementary file 1 — Supplementary Information. [file 41598_2021_4277_MOESM1_ESM.docx]

Differential impact of government lockdown policies on reducing air pollution levels and related mortality in Europe

# Rochelle Schneider1,2,3,4*, Pierre Masselot1, Ana M. Vicedo-Cabrera5,6, Francesco Sera1,7, Marta Blangiardo8, Chiara Forlani8, John Douros9, Oriol Jorba10, Mario Adani11, Rostislav Kouznetsov12,13, Florian Couvidat 14, Joaquim Arteta15, Blandine Raux14, Marc Guevara10, Augustin Colette14, Jérôme Barré4, Vincent-Henri Peuch4, and Antonio Gasparrini1,3,16

1 Department of Public Health, Environments and Society, London School of Hygiene & Tropical Medicine, WC1H 9SH, London, United Kingdom

2 European Space Agency, 00044, Frascati, Italy

3 Centre on Climate Change and Planetary Health, London School of Hygiene & Tropical Medicine, WC1H 9SH, London, United Kingdom

4 [European Centre for Medium-Range Weather Forecast, RG2 9AX, Reading, United Kingdom](mailto:corresponding.author@email.example)

5 Institute of Social and Preventive Medicine, University of Bern, 3012, Bern, Switzerland

6 Oeschger Center for Climate Change Research, University of Bern, 3012, Bern, Switzerland

7 Department of Statistics, Computer Science and Applications "G. Parenti", University of Florence, 60550, Florence, Italy

8 MRC Centre for Environment and Health, Department of Epidemiology and Biostatistics, Imperial College

London, London, W2 1NY, United Kingdom

9 Royal Netherlands Meteorological Institute (KNMI), 3731, GA De Bilt,The Netherlands

10 Barcelona Supercomputing Centre, 08034, Barcelona, Spain

11 Italian National Agency for New Technologies, Energy and Sustainable Economic Development (ENEA), 40129, Bologna,Italy

12 Finnish Meteorological Institute (FMI), 00560, Helsinki, Finland

13 A.M. Obukhov Institute for Atmospheric Physics (IAPh), 119017, Moscow, Russia

14 National Institute for Industrial Environment and Risks (INERIS), 60550, Verneuil-en-Halatte, France

15 National Center for Meteorological Research (CNRM), University of Toulouse, Météo-France, CNRS, UMR 3589, 31057, Toulouse, France

16 Centre for Statistical Methodology, London School of Hygiene & Tropical Medicine, WC1E 7HT, London, United Kingdom

*email: [Rochelle.Schneider@lshtm.ac.uk](mailto:Rochelle.Schneider@lshtm.ac.uk)

| ID | Description | Flag | Levels |
| --- | --- | --- | --- |
| C1 | School closing | Yes | 0 - no measures  1 - recommend closing or all schools open with alterations resulting in significant differences compared to non-Covid-19 operations  2 - require closing (only some levels or categories, eg just high school, or just public schools)  3 - require closing all levels |
| C2 | Workplace closing | Yes | 0 - no measures  1 - recommend closing (or recommend work from home)  2 - require closing (or work from home) for some sectors or categories of workers  3 - require closing (or work from home) for all-but-essential workplaces (e.g. grocery stores, doctors) |
| C3 | Cancel public events | Yes | 0 - no measures  1 - recommend cancelling  2 - require cancelling |
| C4 | Restrictions on gathering | Yes | 0 - no restrictions  1 - restrictions on very large gatherings (the limit is above 1000 people)  2 - restrictions on gatherings between 101-1000 people  3 - restrictions on gatherings between 11-100 people  4 - restrictions on gatherings of 10 people or less |
| C5 | Close public transport | Yes | 0 - no measures  1 - recommend closing (or significantly reduce volume/route/means of transport available)  2 - require closing (or prohibit most citizens from using it) |
| C6 | Stay at home requirements | Yes | 0 - no measures  1 - recommend not leaving house  2 - require not leaving house with exceptions for daily exercise, grocery shopping, and 'essential' trips  3 - require not leaving house with minimal exceptions (eg allowed to leave once a week, or only one person can leave at a time, etc) |
| C7 | Restriction on internal movement | Yes | 0 - no measures  1 - recommend not to travel between regions/cities  2 - internal movement restrictions in place |
| C8 | International travel controls | No | 0 - no restrictions  1 - screening arrivals  2 - quarantine arrivals from some or all regions  3 - ban arrivals from some regions  4 - ban on all regions or total border closure |
| H1 | Public information campaigns | Yes | 0 - no Covid-19 public information campaign  1 - public officials urging caution about Covid-19  2- coordinated public information campaign (eg across traditional and social media) |

Table **A1.** Description of policy indicators used in computing the Stringency Index (SI). The maximum level reported in the fourth column is the value used to standardize the indicators before computing the SI. This original source of this table can be found on the OxCGRT webpage^1^.

| City | Country | SI  Max | NO_2_ | O_3_ | PM_2.5_ | PM_10_ |
| --- | --- | --- | --- | --- | --- | --- |
| Amsterdam | Netherlands | 79.63 | -5.8 (-6.2 ; -5.3) | 2.5 (2.0 ; 3.1) | -1.8 (-2.0 ; -1.6) | -2.0 (-2.2 ; -1.8) |
| Ankara | Turkey | 77.78 | -6.7 (-7.5 ; -6.0) | -1.6 (-2.3 ; -0.9) | -0.3 (-0.5 ; 0.0) | -0.5 (-0.8 ; -0.2) |
| Athens | Greece | 84.26 | -10.2 (-10.7 ; -9.7) | 0.6 (0.1 ; 1.1) | -1.6 (-1.8 ; -1.4) | -2.0 (-2.2 ; -1.8) |
| Barcelona | Spain | 85.19 | -8.7 (-9.1 ; -8.2) | -0.2 (-0.7 ; 0.3) | -1.8 (-2.0 ; -1.6) | -2.1 (-2.3 ; -1.9) |
| Belgrade | Serbia | 100 | -1.2 (-2.2 ; -0.2) | -2.3 (-3.6 ; -1.1) | -0.4 (-0.8 ; -0.0) | -0.5 (-0.9 ; -0.1) |
| Berlin | Germany | 76.85 | -3.3 (-4.1 ; -2.6) | 0.2 (-0.5 ; 1.0) | -1.3 (-1.6 ; -1.1) | -1.5 (-1.8 ; -1.2) |
| Bern | Switzerland | 73.15 | -4.9 (-5.7 ; -4.0) | -2.3 (-3.1 ; -1.6) | -3.0 (-3.3 ; -2.7) | -3.3 (-3.7 ; -3.0) |
| Birmingham | United Kingdom | 75.93 | -7.3 (-8.2 ; -6.5) | 2.5 (1.7 ; 3.3) | -2.5 (-2.8 ; -2.1) | -2.7 (-3.1 ; -2.3) |
| Bratislava | Slovakia | 87.04 | -2.9 (-3.3 ; -2.4) | -1.4 (-1.9 ; -0.9) | -1.1 (-1.2 ; -0.9) | -1.2 (-1.4 ; -1.0) |
| Brussels | Belgium | 81.48 | -8.0 (-8.4 ; -7.7) | 3.0 (2.6 ; 3.4) | -1.9 (-2.1 ; -1.7) | -2.1 (-2.3 ; -2.0) |
| Bucharest | Romania | 87.04 | -3.1 (-3.5 ; -2.6) | -1.5 (-2.0 ; -0.9) | -0.6 (-0.8 ; -0.4) | -0.8 (-0.9 ; -0.6) |
| Budapest | Hungary | 76.85 | -3.4 (-4.1 ; -2.8) | -2.0 (-2.7 ; -1.4) | -0.9 (-1.2 ; -0.7) | -1.1 (-1.4 ; -0.8) |
| Cologne | Germany | 76.85 | -6.0 (-6.6 ; -5.3) | 1.5 (0.8 ; 2.2) | -2.3 (-2.6 ; -2.1) | -2.6 (-2.9 ; -2.3) |
| Copenhagen | Denmark | 72.22 | -4.7 (-6.0 ; -3.4) | 2.6 (1.4 ; 3.8) | -1.3 (-1.7 ; -0.8) | -1.5 (-2.0 ; -1.0) |
| Dublin | Ireland | 90.74 | -2.9 (-3.4 ; -2.4) | 0.3 (-0.3 ; 0.9) | -0.5 (-0.7 ; -0.3) | -0.6 (-0.8 ; -0.4) |
| Hamburg | Germany | 76.85 | -4.3 (-5.0 ; -3.6) | 1.4 (0.7 ; 2.2) | -1.4 (-1.7 ; -1.2) | -1.6 (-1.9 ; -1.3) |
| Helsinki | Finland | 60.19 | -2.1 (-3.7 ; -0.6) | 0.6 (-1.1 ; 2.3) | -0.4 (-1.0 ; 0.1) | -0.6 (-1.1 ; -0.0) |
| Lisbon | Portugal | 87.96 | -6.8 (-7.1 ; -6.4) | 0.6 (0.1 ; 1.0) | -2.7 (-2.9 ; -2.6) | -3.6 (-3.8 ; -3.5) |
| Ljubljana | Slovenia | 89.81 | -2.6 (-3.1 ; -2.1) | -3.1 (-3.7 ; -2.5) | -1.0 (-1.2 ; -0.8) | -1.1 (-1.4 ; -0.9) |
| London | United Kingdom | 75.93 | -9.0 (-10.1 ; -7.9) | 2.9 (1.9 ; 4.0) | -2.6 (-3.0 ; -2.2) | -2.9 (-3.4 ; -2.5) |
| Luxembourg | Luxembourg | 79.63 | -4.8 (-5.3 ; -4.3) | -0.8 (-1.4 ; -0.2) | -2.5 (-2.7 ; -2.3) | -2.7 (-2.9 ; -2.5) |
| Lyon | France | 87.96 | -4.9 (-5.3 ; -4.5) | -2.1 (-2.5 ; -1.6) | -1.2 (-1.4 ; -1.1) | -1.4 (-1.6 ; -1.2) |
| Madrid | Spain | 85.19 | -9.1 (-9.6 ; -8.7) | -1.2 (-1.7 ; -0.7) | -1.2 (-1.4 ; -1.0) | -1.5 (-1.7 ; -1.3) |
| Marseille | France | 87.96 | -2.3 (-2.7 ; -1.9) | -3.2 (-3.6 ; -2.7) | -0.8 (-0.9 ; -0.6) | -0.9 (-1.0 ; -0.7) |
| Milan | Italy | 93.52 | -7.9 (-8.5 ; -7.3) | 2.1 (1.4 ; 2.9) | -2.6 (-2.8 ; -2.4) | -2.8 (-3.0 ; -2.5) |
| Monaco | France | 87.96 | -1.9 (-2.3 ; -1.5) | -4.1 (-4.6 ; -3.7) | -0.9 (-1.0 ; -0.7) | -0.9 (-1.1 ; -0.8) |
| Munich | Germany | 76.85 | -4.6 (-5.3 ; -3.9) | -1.4 (-2.1 ; -0.7) | -2.2 (-2.4 ; -1.9) | -2.4 (-2.7 ; -2.1) |
| Naples | Italy | 93.52 | -5.8 (-6.4 ; -5.2) | 1.3 (0.5 ; 2.0) | -1.1 (-1.3 ; -0.8) | -1.1 (-1.4 ; -0.9) |
| Nicosia | Cyprus | 94.44 | -1.1 (-1.6 ; -0.6) | -3.5 (-4.2 ; -2.9) | -0.4 (-0.6 ; -0.2) | -0.5 (-0.7 ; -0.2) |
| Oslo | Norway | 79.63 | -1.9 (-2.5 ; -1.3) | 0.8 (0.2 ; 1.5) | -0.5 (-0.7 ; -0.2) | -0.6 (-0.8 ; -0.3) |
| Paris | France | 87.96 | -7.0 (-7.9 ; -6.2) | 0.2 (-0.7 ; 1.1) | -1.4 (-1.7 ; -1.1) | -1.7 (-2.0 ; -1.3) |
| Prague | Czech Republic | 82.41 | -3.6 (-4.1 ; -3.0) | 0.6 (-0.1 ; 1.2) | -1.4 (-1.6 ; -1.1) | -1.4 (-1.7 ; -1.2) |
| Pristina | Kosovo | 92.59 | -2.0 (-2.6 ; -1.4) | -1.6 (-2.3 ; -0.8) | -0.4 (-0.6 ; -0.2) | -0.5 (-0.7 ; -0.2) |
| Reykjavik | Iceland | 53.7 | -3.9 (-7.2 ; -0.6) | 0.0 (-2.9 ; 2.9) | -0.7 (-1.9 ; 0.5) | -0.6 (-2.1 ; 0.8) |
| Riga | Latvia | 65.74 | -1.5 (-3.0 ; -0.1) | -0.2 (-1.5 ; 1.0) | -0.4 (-0.8 ; 0.1) | -0.4 (-0.9 ; 0.1) |
| Rome | Italy | 93.52 | -4.8 (-5.3 ; -4.2) | -0.4 (-1.0 ; 0.3) | -1.1 (-1.3 ; -0.9) | -1.2 (-1.4 ; -1.0) |
| Sarajevo | Bosnia and Herzegovina | 92.59 | -0.6 (-1.2 ; -0.0) | -2.4 (-3.1 ; -1.7) | -0.3 (-0.5 ; -0.1) | -0.3 (-0.6 ; -0.1) |
| Sofia | Bulgaria | 73.15 | -3.7 (-4.7 ; -2.8) | -1.8 (-2.6 ; -1.0) | -0.7 (-1.0 ; -0.4) | -0.8 (-1.1 ; -0.5) |
| Stockholm | Sweden | 46.3 | -3.7 (-7.2 ; -0.2) | 0.2 (-2.7 ; 3.2) | -0.8 (-1.8 ; 0.3) | -1.1 (-2.4 ; 0.2) |
| Tallinn | Estonia | 77.78 | -1.4 (-2.1 ; -0.7) | -0.1 (-0.8 ; 0.6) | -0.3 (-0.6 ; 0.0) | -0.3 (-0.6 ; 0.0) |
| Tirana | Albania | 89.81 | -2.4 (-2.9 ; -2.0) | -2.4 (-2.9 ; -1.9) | -0.7 (-0.8 ; -0.5) | -0.8 (-0.9 ; -0.6) |
| Turin | Italy | 93.52 | -5.9 (-6.4 ; -5.4) | 0.2 (-0.4 ; 0.9) | -2.0 (-2.2 ; -1.8) | -2.2 (-2.4 ; -2.0) |
| Valencia | Spain | 85.19 | -5.1 (-5.5 ; -4.7) | -2.2 (-2.6 ; -1.7) | -1.3 (-1.5 ; -1.2) | -1.6 (-1.8 ; -1.4) |
| Vienna | Austria | 85.19 | -3.3 (-3.8 ; -2.8) | -1.2 (-1.7 ; -0.6) | -1.3 (-1.4 ; -1.1) | -1.4 (-1.6 ; -1.2) |
| Vilnius | Lithuania | 87.04 | -1.8 (-2.1 ; -1.4) | -0.4 (-0.9 ; 0.0) | -0.4 (-0.6 ; -0.3) | -0.5 (-0.7 ; -0.3) |
| Warsaw | Poland | 83.33 | -3.2 (-3.7 ; -2.6) | -0.2 (-0.8 ; 0.4) | -0.8 (-1.0 ; -0.6) | -0.9 (-1.1 ; -0.7) |
| Zagreb | Croatia | 96.3 | -1.7 (-2.4 ; -1.1) | -3.3 (-4.1 ; -2.5) | -0.7 (-0.9 ; -0.4) | -0.8 (-1.1 ; -0.6) |
| TOTAL | | 100 | -4.4 (-5.6 ; -3.2) | -0.3 (-1.3 ; 0.6) | -1.0 (-1.6 ; -0.4) | -1.2 (-1.9 ; -0.4) |

Table **A2**. Estimated (mean) and 95% credible limits (low, high) of change in concentration of the air pollutants (µg/m^3^) for an 80% score of the Stringency Index. The chosen threshold of 80% represents the maximum value reached by most of the 47 cities. The column SI Max displays the maximum SI value reached by city

| Model names | Operated by |
| --- | --- |
| CHIMERE^2^ | INERIS (Institut National de l’Environnement Industriel et des Risques) France |
| LOTOS-EUROS^3^ | KNMI (Koninklijk Nederlands Meteorologisch Instituut) the Netherlands |
| MOCAGE^4^ | Meteo-France (France) |
| SILAM^5^ | FMI (Ilmatieteen Laitos) Finland |
| MINNI ^6^ | ENEA (Energy and Sustainable Economic Development), Italy |
| MONARCH^7^ | BSC (Barcelona Supercomputing Centre), Spain |

Table **A3**. Individual models contributing to the CAMS ENSEMBLE.


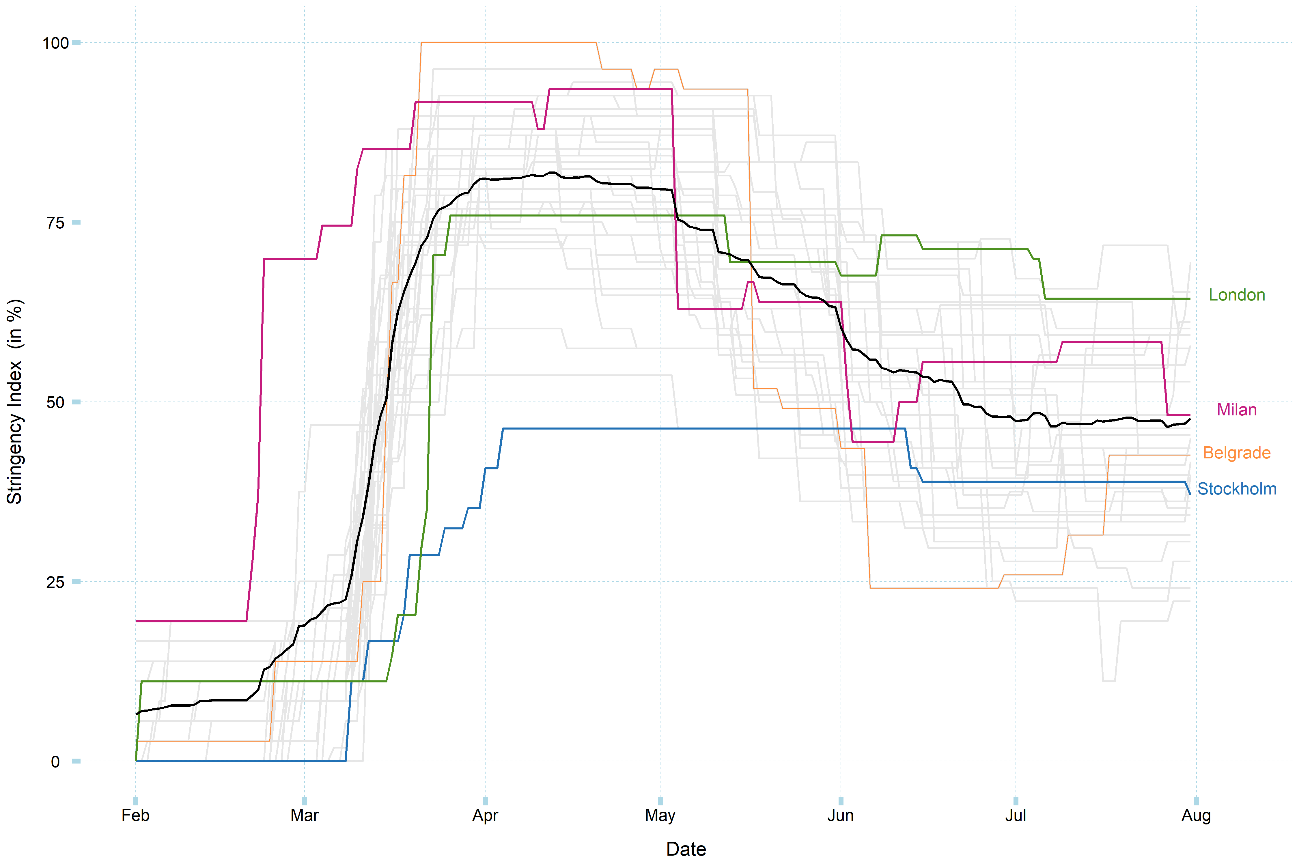


Figure **A1**. Level of SI (policy strictness) over the study period for 47 cities (solid light grey lines) and their average (solid thick black line). Figure created using R software, version 4.0.3^8^

| City name | Country | Total deaths | Excess deaths by pollutant-specific using only the **BAU** scenario concentrations | | | |
| --- | --- | --- | --- | --- | --- | --- |
|  |  |  | NO_2_ | O_3_ | PM_2.5_ | PM_10_ |
| Amsterdam | Netherlands | 3,899 | 32.9 (26.1 ; 39.3) | 53.9 (35.9 ; 71.3) | 22.0 (19.1 ; 24.9) | 21.1 (18.9 ; 23.5) |
| Ankara | Turkey | 7,131 | 65.7 (52.1 ; 78.4) | 112.7 (75.1 ; 149.0) | 92.6 (80.5 ; 104.7) | 85.0 (76.2 ; 94.4) |
| Athens | Greece | 17,842 | 244.1 (193.9 ; 291.2) | 301.2 (200.6 ; 398.1) | 148.5 (129.0 ; 168.0) | 155.4 (139.1 ; 172.4) |
| Barcelona | Spain | 16,670 | 218.8 (173.7 ; 261.0) | 258.6 (172.2 ; 341.8) | 151.4 (131.5 ; 171.2) | 139.6 (125.0 ; 155.0) |
| Belgrade | Serbia | 6,979 | 18.6 (14.8 ; 22.3) | 108.9 (72.5 ; 143.9) | 59.7 (51.9 ; 67.5) | 49.9 (44.6 ; 55.3) |
| Berlin | Germany | 16,108 | 73.3 (58.2 ; 87.6) | 234.2 (155.9 ; 309.6) | 80.7 (70.1 ; 91.3) | 70.3 (62.9 ; 78.0) |
| Bern | Switzerland | 898 | 3.2 (2.5 ; 3.8) | 13.9 (9.3 ; 18.4) | 4.3 (3.8 ; 4.9) | 3.7 (3.4 ; 4.2) |
| Birmingham | United Kingdom | 8,984 | 49.5 (39.3 ; 59.1) | 126.5 (84.2 ; 167.3) | 49.3 (42.8 ; 55.8) | 46.7 (41.8 ; 51.8) |
| Bratislava | Slovakia | 1,684 | 6.8 (5.4 ; 8.1) | 26.0 (17.3 ; 34.3) | 9.8 (8.5 ; 11.1) | 7.7 (6.9 ; 8.6) |
| Brussels | Belgium | 5,077 | 43.3 (34.4 ; 51.7) | 71.5 (47.6 ; 94.5) | 35.0 (30.4 ; 39.6) | 30.4 (27.3 ; 33.8) |
| Bucharest | Romania | 10,704 | 73.4 (58.3 ; 87.7) | 164.9 (109.8 ; 217.9) | 95.3 (82.8 ; 107.8) | 79.4 (71.1 ; 88.1) |
| Budapest | Hungary | 10,783 | 61.7 (49.0 ; 73.7) | 167.3 (111.4 ; 221.1) | 82.9 (72.1 ; 93.8) | 69.0 (61.8 ; 76.6) |
| Cologne | Germany | 8,161 | 77.3 (61.3 ; 92.2) | 115.4 (76.8 ; 152.6) | 53.8 (46.7 ; 60.8) | 47.4 (42.5 ; 52.7) |
| Copenhagen | Denmark | 5,764 | 26.7 (21.2 ; 31.9) | 79.3 (52.8 ; 104.8) | 24.5 (21.3 ; 27.8) | 24.9 (22.3 ; 27.6) |
| Dublin | Ireland | 2,960 | 13.9 (11.0 ; 16.6) | 41.0 (27.3 ; 54.2) | 12.5 (10.9 ; 14.2) | 14.4 (12.9 ; 15.9) |
| Hamburg | Germany | 7,854 | 61.3 (48.7 ; 73.2) | 107.4 (71.5 ; 142.0) | 41.3 (35.8 ; 46.7) | 38.6 (34.6 ; 42.9) |
| Helsinki | Finland | 3,320 | 12.2 (9.7 ; 14.6) | 45.6 (30.4 ; 60.3) | 9.7 (8.4 ; 11.0) | 10.3 (9.2 ; 11.4) |
| Lisbon | Portugal | 9,775 | 64.8 (51.5 ; 77.4) | 151.3 (100.7 ; 199.9) | 83.0 (72.1 ; 93.9) | 85.8 (76.8 ; 95.2) |
| Ljubljana | Slovenia | 1,023 | 5.5 (4.4 ; 6.6) | 16.3 (10.8 ; 21.5) | 9.7 (8.4 ; 10.9) | 7.2 (6.5 ; 8.0) |
| London | United Kingdom | 26,966 | 222.0 (176.2 ; 265.0) | 380.1 (253.0 ; 502.5) | 162.0 (140.7 ; 183.3) | 152.6 (136.6 ; 169.4) |
| Luxembourg | Luxembourg | 424 | 1.7 (1.4 ; 2.1) | 6.3 (4.2 ; 8.3) | 2.2 (1.9 ; 2.5) | 1.9 (1.7 ; 2.1) |
| Lyon | France | 3,993 | 31.8 (25.2 ; 37.9) | 61.8 (41.1 ; 81.6) | 26.5 (23.1 ; 30.0) | 23.2 (20.8 ; 25.7) |
| Madrid | Spain | 17,256 | 152.5 (121.1 ; 182.0) | 274.6 (182.9 ; 362.9) | 114.3 (99.3 ; 129.3) | 104.7 (93.7 ; 116.2) |
| Marseille | France | 4,039 | 16.9 (13.4 ; 20.2) | 70.8 (47.1 ; 93.5) | 21.0 (18.2 ; 23.8) | 20.4 (18.2 ; 22.6) |
| Milan | Italy | 14,221 | 151.2 (120.1 ; 180.4) | 250.5 (166.9 ; 331.0) | 169.0 (147.0 ; 191.1) | 125.4 (112.3 ; 139.2) |
| Monaco | France | 320 | 1.0 (0.8 ; 1.2) | 5.7 (3.8 ; 7.5) | 1.6 (1.4 ; 1.8) | 1.4 (1.2 ; 1.5) |
| Munich | Germany | 5,979 | 37.4 (29.7 ; 44.7) | 93.0 (61.9 ; 123.0) | 32.9 (28.6 ; 37.3) | 27.5 (24.6 ; 30.5) |
| Naples | Italy | 13,563 | 125.7 (99.8 ; 150.1) | 235.0 (156.5 ; 310.5) | 107.2 (93.2 ; 121.3) | 103.5 (92.7 ; 114.9) |
| Nicosia | Cyprus | 757 | 1.8 (1.4 ; 2.1) | 13.7 (9.2 ; 18.2) | 4.4 (3.8 ; 5.0) | 5.2 (4.6 ; 5.7) |
| Oslo | Norway | 2,241 | 13.0 (10.3 ; 15.5) | 29.9 (19.9 ; 39.6) | 9.4 (8.2 ; 10.7) | 7.8 (7.0 ; 8.7) |
| Paris | France | 30,743 | 291.6 (231.5 ; 348.0) | 436.3 (290.5 ; 576.9) | 216.1 (187.8 ; 244.5) | 192.0 (171.9 ; 213.2) |
| Prague | Czech Republic | 5,355 | 23.8 (18.9 ; 28.4) | 80.9 (53.9 ; 106.9) | 33.8 (29.3 ; 38.2) | 26.5 (23.7 ; 29.4) |
| Pristina | Kosovo | NA | NA (NA ; NA) | NA (NA ; NA) | NA (NA ; NA) | NA (NA ; NA) |
| Reykjavik | Iceland | 591 | 1.0 (0.8 ; 1.2) | 8.1 (5.4 ; 10.7) | 1.1 (1.0 ; 1.3) | 1.4 (1.2 ; 1.5) |
| Riga | Latvia | 3,811 | 12.8 (10.1 ; 15.3) | 51.4 (34.2 ; 68.0) | 21.2 (18.4 ; 24.0) | 18.6 (16.6 ; 20.6) |
| Rome | Italy | 10,969 | 72.8 (57.8 ; 87.0) | 188.4 (125.5 ; 249.0) | 83.3 (72.4 ; 94.2) | 78.0 (69.9 ; 86.6) |
| Sarajevo | Bosnia and Herzegovina | 1,974 | 2.9 (2.3 ; 3.5) | 30.2 (20.1 ; 39.9) | 14.1 (12.3 ; 16.0) | 12.2 (10.9 ; 13.5) |
| Sofia | Bulgaria | 5,382 | 18.9 (15.0 ; 22.6) | 81.8 (54.4 ; 108.1) | 44.4 (38.6 ; 50.2) | 39.0 (34.9 ; 43.3) |
| Stockholm | Sweden | 4,535 | 16.4 (13.0 ; 19.6) | 62.8 (41.8 ; 83.0) | 13.0 (11.3 ; 14.7) | 12.9 (11.5 ; 14.3) |
| Tallinn | Estonia | 1,695 | 6.0 (4.7 ; 7.1) | 23.2 (15.4 ; 30.6) | 5.4 (4.7 ; 6.1) | 4.7 (4.2 ; 5.3) |
| Tirana | Albania | 2,086 | 8.2 (6.5 ; 9.8) | 35.0 (23.3 ; 46.2) | 15.5 (13.5 ; 17.5) | 14.6 (13.0 ; 16.2) |
| Turin | Italy | 6,899 | 53.5 (42.5 ; 63.9) | 119.5 (79.6 ; 157.9) | 80.0 (69.6 ; 90.5) | 59.4 (53.2 ; 65.9) |
| Valencia | Spain | 6,400 | 38.7 (30.7 ; 46.2) | 107.1 (71.3 ; 141.6) | 41.9 (36.4 ; 47.4) | 40.5 (36.3 ; 45.0) |
| Vienna | Austria | 8,226 | 46.5 (36.9 ; 55.5) | 127.2 (84.7 ; 168.1) | 45.8 (39.8 ; 51.9) | 37.4 (33.5 ; 41.5) |
| Vilnius | Lithuania | 2,107 | 4.4 (3.5 ; 5.3) | 28.3 (18.8 ; 37.4) | 8.3 (7.2 ; 9.4) | 6.8 (6.1 ; 7.5) |
| Warsaw | Poland | 9,423 | 52.8 (41.9 ; 63.0) | 134.2 (89.3 ; 177.4) | 73.1 (63.6 ; 82.8) | 59.6 (53.4 ; 66.2) |
| Zagreb | Croatia | 3,680 | 14.3 (11.3 ; 17.1) | 58.6 (39.0 ; 77.5) | 27.0 (23.4 ; 30.5) | 22.4 (20.1 ; 24.9) |
| TOTAL | | 339,251 | 2,572.9  (2,042.3 ; 3,070.9) | 5,190.0  (3,455.9 ; 6,860.7) | 2,440.6  (2,120.8 ; 2,761.2) | 2,186.1  (1,957.5 ; 2,427.1) |

Table **A4**. The sample of 46 cities (except Pristina (Kosovo)) selected from the European CAMS Air Quality information webpage. Reported are: total deaths and an estimate of the death burden of each pollutant under BAU scenario. The total deaths were estimated for each city using the crude death rate, population, and the number of days comprising the study period (February-July). The excess deaths were estimated for each pollutant and city based on Equation 3, replacing $y_{it}$ by the corresponding BAU-only concentration time series.

# References

1. OxCGRT/covid-policy-tracker. *GitHub* https://github.com/OxCGRT/covid-policy-tracker.

2. Mailler, S. *et al.* CHIMERE-2017: from urban to hemispheric chemistry-transport modeling. *Geosci. Model Dev.* **10**, 2397–2423 (2017).

3. Manders, A. M. M. *et al.* Curriculum vitae of the LOTOS–EUROS (v2.0) chemistry transport model. *Geosci. Model Dev.* **10**, 4145–4173 (2017).

4. Guth, J., Josse, B., Marécal, V., Joly, M. & Hamer, P. First implementation of secondary inorganic aerosols in the MOCAGE version R2.15.0 chemistry transport model. *Geosci. Model Dev.* **9**, 137–160 (2016).

5. Sofiev, M. *et al.* *Construction of an Eulerian atmospheric dispersion model based on the advection algorithm of M. Galperin: dynamic cores v.4 and 5 of SILAM v.5.5*. https://gmd.copernicus.org/preprints/8/2905/2015/gmdd-8-2905-2015.pdf (2015) doi:10.5194/gmdd-8-2905-2015.

6. AMS-MINNI national air quality simulation on Italy for the calendar year 2015. Annual air quality simulation of MINNI Atmospheric Modelling System: results for the calendar year 2015 and comparison with observed data. https://iris.enea.it/handle/20.500.12079/52259#.YSI93IhKhPY.

7. Badia, A. *et al.* Description and evaluation of the Multiscale Online Nonhydrostatic AtmospheRe CHemistry model (NMMB-MONARCH) version 1.0: gas-phase chemistry at global scale. *Geosci. Model Dev.* **10**, 609–638 (2017).

8. R: The R Project for Statistical Computing. https://www.r-project.org/.
